# Acknowledgement of manuscript reviewers

**DOI:** 10.1186/1754-1611-7-4

**Published:** 2013-03-26

**Authors:** Mark Eiteman

**Affiliations:** 1 College of Engineering, University of Georgia, Athens, GA 30602, United States of America

## Abstract

**Contributing reviewers:**

The editors of the Journal of Biological Engineering thank all our reviewers who have contributed to the journal in volume 5 (2011) and volume 6 (2012).

## 

Stephen Abedon

United States of America

Yagut Allahverdiyeva

Finland

John Anderson

United States of America

James Baish

United States of America

Rekha Balachandran

United States of America

Geoff Baldwin

United Kingdom

Ipsita Banerjee

United States of America

Philip Barnes

United States of America

Carl Batt

United States of America

Travis Bayer

United Kingdom

Danielle Bellmer

United States of America

Matthew Bennett

United States of America

Leonidas Bleris

United States of America

Nils Bluthgen

Germany

Frank Bosse

Germany

Pierre-Yves Bourguignon

Germany

Kevin Braeckmans

Belgium

Gail Brion

United States of America

Eric Brouzes

United States of America

Steven Brown

United States of America

Dennis Brown

United States of America

A. Malcolm Campbell

United States of America

Lisa Casanova

United States of America

Ken Chan

Hong Kong

Deepak Chandran

United States of America

Chih Chien

Taiwan

Wei-Chun Chin

United States of America

Hae Woon Choi

South Korea

Jin-Woo Choi

United States of America

Patrick Cirino

United States of America

Andrew Coates

United Kingdom

Rory Coffey

Ireland

Teodoro Córdova

Mexico

Sophie Demoustier-Champagne

Belgium

Douglas Densmore

United States of America

Pavel Dibrov

Canada

Vera Doroshenko

Russian Federation

Mary Dunlop

United States of America

Mark Eiteman

United States of America

Tom Ellis

United Kingdom

Drew Endy

United States of America

Daniel Euhus

United States of America

Stephen Fong

United States of America

Paul Freemont

United Kingdom

Cinzia Freschi

Italy

Steve Furber

United Kingdom

Munia Ganguli

India

Max Garzon

United States of America

Eric Gaucher

United States of America

R. Christopher Geiger

United States of America

Charles Gersbach

United States of America

George Gillies

United States of America

David Graham

United States of America

Haigang Gu

United States of America

Mark Haidekker

United States of America

Xiaoming He

United States of America

Nathan Hillson

United States of America

Christie Howard

United States of America

Shyh-shin Hwang

Taiwan

Lorens Imhof

Germany

Pieter Jacobs

United States of America

Michael Jewett

United States of America

Philip Johnson

United States of America

Patrik Raymond Jones

Finland

Han Jong-In

South Korea

Emil P Kartalov

United States of America

Robert Kelly

United States of America

Jungkyu Kim

United States of America

Mattheos Koffas

United States of America

Werner JH Koopman

Netherlands

Pamela Kreeger

United States of America

Natalie Kuldell

United States of America

S. C. Kundu

India

Soonjo Kwon

United States of America

Thomas Landrain

France

Helene Langevin

United States of America

Michelle LaPlaca

United States of America

Gaetan Laroche

Canada

Sang Yup Lee

South Korea

James Lee

United States of America

Stephen Lee

United States of America

Petros Lenas

Spain

Yin Li

China

Yanna Liang

United States of America

Marybeth Lima

United States of America

Jian Lin

United States of America

Tom Lindstrom

Sweden

Andreas Linninger

United States of America

Tim Lu

United States of America

Toshinari Maeda

Japan

Abdul Mannan

Pakistan

Leidong Mao

United States of America

Mario Andrea Marchisio

Switzerland

Ian Marison

Ireland

Jose Luis Martinez-Carrillo

Mexico

Marty Matlock

United States of America

Roberto Mazzoli

Italy

Kara McCloskey

United States of America

Todd Monroe

United States of America

Suchetana Mukhopadhyay

United States of America

Christine Mulvey

United States of America

Pablo I Nikel

Argentina

Goutam Nistala

United States of America

Vincent Noireaux

United States of America

Angela Pannier

United States of America

Jin-Byung Park

South Korea

Daniel Park

United States of America

Tom Pearson

United States of America

Kristala Prather

United States of America

Michael Raghunath

Singapore

Ann Rajnicek

United Kingdom

Ramaraja Ramasamy

United States of America

Balaji Rao

United States of America

Jakob Reiser

United States of America

Tom Richard

United States of America

Mark Riley

United States of America

Phillip Roberts

United States of America

Kenneth Robinson

United States of America

Roberto Rodriguez

Mexico

Jeremy Rogers

United States of America

Jennifer Ross

United States of America

Roger Ruan

United States of America

Armenio Santos

Brazil

Frank Sargent

United Kingdom

Ryan Senger

United States of America

Rakesh Sharma

India

Reshma Shetty

United States of America

Pamela Silver

United States of America

Istvan Siro

Hungary

Sean Sleight

United States of America

Chinmay Soman

United States of America

Hao Song

Singapore

Oliver Spadiut

Austria

Friedrich Srienc

United States of America

Gene Stevens

United States of America

Michael Storrie-Lombardi

United States of America

Charles Suh

United States of America

Kahp-Yang Suh

South Korea

Lianhong Sun

United States of America

Dong Sun

United States of America

Jeffrey Tabor

United States of America

Maryam Tabrizian

Canada

Ralf Takors

Germany

Timothy Taylor

United States of America

Glynn Tillman

United States of America

George Tsibidis

Greece

Andrey Turchinovich

Germany

Sofie Van Den Hende

Belgium

Brahm Verma

United States of America

Junwei Wang

China

Wilfried Weber

Germany

Lisa Wilken

United States of America

Hai Yan

China

Habibollah Younesi

Iran

Jiajia Yuan

Australia

Kechun Zhang

United States of America

Shusheng Zhang

China

